# Placebo and nocebo phenomena in schizophrenia spectrum disorders: a narrative review on current knowledge and potential future directions

**DOI:** 10.1017/S0033291725100901

**Published:** 2025-07-18

**Authors:** James A. Waltz, Sherry D. Pujji, Luana Colloca

**Affiliations:** 1 Maryland Psychiatric Research Center (MPRC), Department of Psychiatry, University of Maryland School of Medicine, Baltimore, MD, USA; 2 Department of Pain and Translational Symptom Science, Placebo Beyond Opinions (PBO) Center, University of Maryland School of Nursing, Baltimore, MD, USA

**Keywords:** Bayesian, conditioning, negative symptoms, positive symptoms, predictive coding, psychosis, randomized clinical trials (RCTs)

## Abstract

The experience of psychosis in schizophrenia spectrum disorders involves significant distress and functional impairment, contributing to immense social and economic costs. Current gold standard treatment guidelines emphasize the use of antipsychotic medications, though responses to these treatments vary widely, with the potential for detrimental side effects. However, increasing placebo responses in randomized controlled trials since the 1960s complicate the development of new medications. Elevated placebo responses are common in psychiatric populations, including those with psychosis, and are influenced by individual beliefs and prior experiences. Despite extensive research on placebo mechanisms in conditions such as depression and pain, little is known about mechanisms of these effects in psychosis. This narrative review examines the predictors and belief formation processes underlying placebo and nocebo phenomena in psychosis. We discuss features of randomized controlled trials for antipsychotic medications, individual symptom heterogeneity, and contextual factors. Findings related to placebo effects for motivation and cognition-enhancing drugs are also discussed. We then consider the possibility that theories of predictive coding and aberrant salience provide explanation for aspects of both placebo effects and schizophrenia spectrum symptoms. The role of outcome expectations broadly and in the context of reward processing is considered. We conclude with some recommendations for future placebo research in psychosis, emphasizing the diversity of placebo effects, assessment concerns, cultural considerations, and methodological aspects. Future multidisciplinary research is required to further elucidate placebo effects in schizophrenia spectrum disorders.

## Introduction

The experience of schizophrenia spectrum disorders (SSDs) involves symptoms and impairments associated with enormous social and financial costs (Ali, Santomauro, Ferrari, & Charlson, 2023; American Psychiatric Association (APA), 2022; Kadakia et al., 2022; Oh, Waldman, Stickley, DeVylder, & Koyanagi, 2019; Stubbs et al., 2016). While current psychosis treatment guidelines often prioritize the use of antipsychotic medications, the literature on treatment responses is mixed. There appears to be evidence for both broad symptom improvements (McCutcheon et al., 2021) and variations of antipsychotic medication responsiveness (Case et al., 2011; Stroup, 2007). Furthermore, antipsychotic medications also often have detrimental side effects, including movement disorders and weight gain (Ceraso et al., 2020; Doane et al., 2020). Importantly – in addition to multitude of other treatment development challenges, such as the presence of immense heterogeneity in psychosis symptom profiles (APA, 2022; Tsuang & Faraone, 1995) – the use and development of medications to manage psychosis has been complicated by trends pointing toward increasing placebo responses and decreasing responses to active treatments in randomized controlled trials (RCTs; Hird, Diederen, Leucht, Jensen, & McGuire, 2023; Leucht et al., 2017). The reasons for increasing placebo responses – apparent improvements on the relevant outcome measure in the placebo group of a clinical trial – are not fully understood. While much of the magnitudes of placebo responses observed in RCTs with psychiatric medications can be attributed to genuine placebo *effects* in individuals (defined as advantageous outcomes in response to inactive treatments that can influence an individual’s beliefs beyond that of any treatment effects; Neogi & Colloca, 2023), many other factors influence the size of these responses. Furthermore, the degree to which reports of medication side effects reflect nocebo effects (defined as subjective experiences of aversive outcomes in response to inactive treatments) is not known (Atlas, 2023; Colloca et al., 2023). There is some evidence to suggest that the perception of adverse events, consequent to nocebo effects, is related to elevated levels of psychiatric symptoms and may contribute to treatment noncompliance and attrition, in the context of RCTs (Blasini et al., 2017; Wartolowska, 2019). In short, while treatment responses clearly exceed placebo responses in many clinical trials (Taylor, Smith, Gee, & Nielsen, 2012), the last six decades have seen a reduction in *differences* between drug and placebo response effects (Agid et al., 2013; Hird et al., 2023; Leucht et al., 2017).

While much of the literature focuses on profound placebo effects under depressive and pain conditions (Atlas, 2023; Colloca et al., 2023; Murray & Stoessl, 2013), placebo effects appear to vary considerably within and across psychiatric disorders and drug classes (Cao et al., 2021; Murray & Stoessl, 2013). While placebo effect estimates for antipsychotic medications range anywhere from 20 to 70% (Cao et al., 2021; Murray & Stoessl, 2013), those estimates pale in comparison to placebo effect estimates for antidepressant medications, which range from 70 to 90% (Cao et al., 2021). However, it is not known whether specific predictors and belief processes that maintain placebo and nocebo effects in psychosis are the same in other populations. In a recent review, Hird et al. (2023) have suggested that neurocognitive mechanisms, formalized in theories of predictive coding, may provide a useful framework to understand the unique qualities of placebo and nocebo responses, in terms of both the alleviation of psychotic symptoms such as delusions (which are themselves beliefs) and believing that a medicine is actually reducing symptoms (Hird et al., 2023). If we are to isolate treatment effects in future RCTs for psychiatric medications, there is an urgent need to increase our understanding of the origins of placebo and nocebo responses in patients with psychosis (Alphs, Benedetti, Fleischhacker, & Kane, 2012; Hird et al., 2023). That is, developing a better understanding of placebo effects and their predictors may lead to improvements in health care.

In this narrative review, we extend the observations, recommendations, and guidance provided by Hird et al. (2023) by considering additional factors that may modulate placebo effects in psychosis. These include the unique features of antipsychotic medications and RCTs, symptom heterogeneity, and the roles of expectations, insight, reward sensitivity, and other contextual factors. We conclude with specific recommendations for research in psychosis focused on different types of placebo effects, assessment concerns, and cultural considerations.

## Predictors of placebo responses in RCTs for antipsychotic medications in people with SSDs

**Figure 1.**
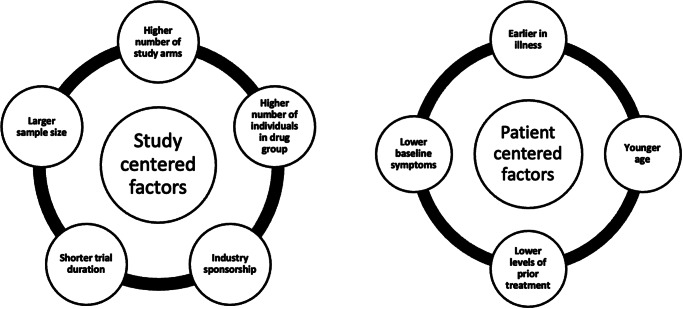
Summary of study- and patient-centered factors influencing placebo effects in SSDs as identified by Hird et al. (2023). This meta-analysis identified several study- and patient-centered factors that may play a role in elevated placebo effects for some individuals with schizophrenia.

Although the specific predictors of placebo responses in antipsychotic medication trials have yet to be purposefully and clearly examined, a number of potential predictors that may guide RCT design and augment clinical practice have been identified (Hird et al., 2023). These predictors have been grouped into study-centered factors (number of study sites, trial duration, sample size, industry sponsorship, and number of individuals in the drug versus placebo group) and patient-centered factors (duration of illness, younger age, prior treatment experiences; Hird et al., 2023; see Figure 1).

In terms of study-centered factors, greater numbers of participants in the placebo arm of a trial (relative to the treatment arm) have contributed to increased drug-placebo differences in both antipsychotic and antidepressant drug trials (Mallinckrodt, Zhang, Prucka, & Millen, 2010). The quality, sponsorship, and location of study sites may also partially contribute to the magnitudes of placebo responses (Hird et al., 2023). Study sites for RCTs are subject to a variety of regulations, guidance, and pressures (Bhide, Shah, & Acharya, 2018). Of note, trends of increased placebo responses and decreased treatment effects are especially prevalent in North America (Chen, Wang, Khin, Hung, & Laughren, 2010; Khin, Chen, Yang, Yang, & Laughren, 2012). Industry sponsorship may also increase pressure to obtain desired results and reduce the rigor of specific study arms. Fraguas et al. (2019) argued that industry-sponsored sites may recruit ‘professional patients’ who tend to have less severe baseline symptoms (Rutherford et al., 2014). Yet, research has also found that purposely recruiting individuals with elevated symptom profiles does not add advantages in quality and may create disadvantages (Welge & Keck, 2003). Nonetheless, industry-sponsored trials tend to have elevated placebo responses, relative to non-industry-sponsored trials (Haddad & Correll, 2018; Leucht et al., 2017).

With regard to patient-centered factors, there is mixed support for symptom severity being a predictor of the magnitude of placebo responses (Hird et al., 2023), as some meta-analyses have found support for stronger placebo effects being related to higher baseline symptoms (Agid et al., 2013), and others indicate that stronger placebo effects are associated with *milder* baseline symptoms (Hird et al., 2023; Weimer, Colloca, & Enck, 2015). Furthermore, individuals with higher positive and total SSD symptoms may find the attention received in RCTs or through hospital stabilization to reinforce positive expectancies and thus have greater placebo responses (Agid et al., 2013). Individuals recruited to participate in RCTs may also be subject to practices geared toward altering their expectations of study-related outcomes (Rutherford et al., 2014). Specifically, it has been suggested that placebo research may be subject to the ‘halo effect’ where participants may allow their positive views of research-related staff to influence their expectations (Andrade, 2012; Murray & Stoessl, 2013). Other potential influential factors include the Hawthorne effect (i.e. knowledge of being observed influences observations), Rosenthal effect (i.e. inaccurate perception of improvement over time by raters), and contextual factors such as social support (see Andrade [2012] for detailed discussion).

## Predictors of placebo and nocebo effects in patients with SSDs

In contrast to placebo responses observed in RCTs, placebo effects are mechanistically built on expectations and beliefs regarding the effectiveness of an inactive intervention (Colloca & Barsky, 2020; Colloca et al., 2023). Expectations (priors), specifically outcome-related expectations, appear to be central to placebo effects across psychiatric conditions (Rief & Petrie, 2016). Expectations of improvement can be deduced from study-related factors such as an individual believing that they are assigned to an active RCT treatment group because they know there are many treatment arms and likely only one placebo group, which has been shown to mediate placebo responses (Fraguas et al., 2019). The fact that expectations, consequent to conditioning, may wane over time, due to decay or extinction (Hird et al., 2023), may partially explain why RCTs of longer duration tend to show smaller placebo effects. Agid et al. (2013) noted that individuals may also be aware of intrinsic factors, such as the presence or absence of extrapyramidal side effects, which may influence their group membership expectations, and subsequently, outcome expectations. Research groups have suggested to try to account for individual differences when designing and recruiting for clinical trials to better account for and address placebo responders in RCTs (Agid et al., 2013; Hird et al., 2023). Of note, antipsychotic RCTs rarely, if ever, directly query participants’ expectations (Fraguas et al., 2019); doing so would contribute greatly to our understanding of placebo responses in psychiatric patients.

The Placebo-Reward Hypothesis posits that expectations of benefit may actually cause improvement in RCTs with medications for Parkinson’s disease (de la Fuente-Fernández, 2009). That is, the anticipation of successful treatment (i.e., reward) may inherently boost dopamine and thereby mood (Murray & Stoessl, 2013). The expectation of improvement may itself be antidepressant. Recent research has also found that treatment uncertainty and reward-related learning may modulate placebo effects (Augustat, Endres, & Mueller, 2024). Regarding large placebo effects for antidepressant medications, research has emphasized maximizing positive associations through conditioning to increase reward anticipation (Murray & Stoessl, 2013).

While placebo responses and effects have been linked to psychological and pathophysiological mechanisms (Colloca et al., 2023), the neural and psychological origins of nocebo effects are less-well understood (Atlas, 2023; Colloca et al., 2023). Recent research outside of SSDs has suggested that a range of biological and neurophysiological factors surrounding adverse experiences influences susceptibility to nocebo effects (Grosso et al., 2024). The valences of the *expectations* (positive/optimistic or negative/pessimistic) are likely to influence whether the *effects* of inactive compounds are falsely perceived as positive or negative. That is, positive expectations may be linked to placebo effects, while negative expectations may be linked to nocebo effects (Rief & Petrie, 2016). Coupled with existing priors, sensitivity to reward may bolster positive expectancies associated with placebo effects, as has been observed in placebo analgesia (De Pascalis & Scacchia, 2019). Outcome expectations are inherently linked to reward and punishment sensitivity (Murray & Stoessl, 2013). Finally, while not consistently examined in the literature, it may be possible that individuals with SSDs are particularly sensitive to nocebo effects where they might believe that inactive interventions could be causing them harm. This is especially salient when considering symptoms such as specific types of delusional ideation where individuals may be prone to believing that innocuous stimuli or individuals mean them harm (APA, 2022). Additionally, it is plausible that comorbid mood symptoms increase the likelihood that outcome expectations are negative (Palermo et al., 2019). In any case, much research is needed to further identify the nocebo effects across SSDs and understand their sources.

Individuals with comorbid depressive and psychotic symptoms tend to be less responsive to placebo antidepressants, when compared with individuals who only had depression (Glassman & Roose, 1981). While this suggests that there may be unique processes associated with psychosis that influence the likelihood of placebo responses (and effects, mechanistically speaking), these processes have not yet been clearly identified. For example, reduced levels of insight into symptoms and diagnoses in SSDs may be one potential differentiator (Cao et al., 2021), while differences in outcome expectations may be another (Murray & Stoessl, 2013). However, it is unknown if shared mechanisms that underlie placebo effects are consistent across SSDs and other diagnoses. The ease of identifying reward-related placebo effects in SSDs might be hampered by individuals experiencing symptoms such as delusions as rewarding experiences (Murray & Stoessl, 2013). A better understanding of the importance and value of beliefs and expectations to the individual, as they relate to reward prediction and reward processing, may greatly contribute to placebo response research in SSDs (Petrovic & Sterzer, 2023). Individuals with SSDs may have impairments in multiple aspects of belief formation, including prediction error signaling in reward and value-based decision-making (Sterzer et al., 2019) that may generate differential susceptibility to placebo and nocebo effects.

## Predictive coding theory accounts of placebo effects

Given that placebo *effects* are mechanistically built on expectations and beliefs (Colloca & Barsky, 2020; Colloca et al., 2023), understanding the origins of placebo effects requires the consideration of theories of belief formation. One of the most detailed theories of belief formation, at the present time, is predictive coding (PC) theory, the central tenets of which are tied closely to Bayesian theory (Büchel et al., 2014; Kaptchuk, Hemond, & Miller, 2020). Broadly, PC predicting coding frameworks are built on the idea that perception involves a combination of bottom-up and top-down processing, and consequently, perception can be strongly influenced by expectations (Büchel, Geuter, Sprenger, & Eippert, 2014; Sterzer et al., 2018). That is, PC theory posits that top-down prior beliefs and experiences (priors), coupled with bottom-up sensory information (likelihoods), lead individuals to make inferences about how to navigate their environment (Sterzer et al., 2018). Neurocomputationally, the brain combines priors and likelihoods into posteriors, or an interpretation of current experiences (Petrovic & Sterzer, 2023). Any mismatches between priors and likelihoods result in prediction errors, which may subsequently contribute to changes in one’s internal model of priors to reduce prediction-error-related dissonance (Petrovic & Sterzer, 2023). Predictive coding theory suggests that discrepancies in the weighting of priors and likelihoods are responsible for both SSD symptoms, such as delusions, and placebo effects, perhaps highlighting similarities between the two constructs (Sterzer et al., 2018; Sterzer, Voss, Schlagenhauf, & Heinz, 2019). Computational modeling analyses also suggest that the lower- and higher-order beliefs and expectations inherent in delusions may generate somewhat analogous placebo effects to those of the opioid network when considering pain (Hird et al., 2023).

Several aspects of psychosis may emerge from a reduced ability to integrate bottom-up and top-down signals, including auditory hallucinations, delusions, reinforcement learning impairments, and an altered sense of agency (Petrovic & Sterzer, 2023; Sterzer et al., 2018). Computational PC frameworks may further assist in understanding processes critical to learning and inference, such as the perception of volatility, precision/uncertainty, and the amplification of prediction error signals through learning rates (Hird et al., 2023). There is a large body of work that links hallucinations to overly strong prior beliefs (Corlett et al., 2019), whereas delusions have been associated with the presence of imprecise priors that may or may not be strong in nature (Petrovic & Sterzer, 2023). For example, delusions have been associated with overly strong priors (Baker et al., 2019) and more broadly, overly strong priors might also influence beliefs about treatment effects and lead to placebo or nocebo effects (Grosso et al., 2024; Hird et al., 2023). Conversely, the presence of weaker low-level priors and increased emphasis on higher-level priors may also contribute to delusions and placebo responses, as individuals may be attempting to detect patterns to form expectations (Petrovic & Sterzer, 2023). The *valence* of beliefs about treatment effects might further be influenced by a subject’s degree of paranoia, which has been linked (through PC theory) to beliefs about environmental volatility (Sheffield et al., 2022).

Predictions and certainty appear to influence expectations at different levels, thus lending itself to specific symptom profiles that may be more susceptible to placebo and nocebo effects. For example, delusional beliefs have been explained in terms of an increased weighting of priors (Petrovic & Sterzer, 2023; Sterzer et al., 2018), which may potentially signal susceptibility to placebo effects. Delusional beliefs have also been explained in terms of stronger priors related to *higher-level* beliefs, which compensate for weaker, ambiguity-driven priors at lower levels (Petrovic & Sterzer, 2023). This suggests that in the case of delusions, beliefs about environmental contingencies may be more imprecise, while beliefs about environmental volatility may be more rigid. Preliminary evidence in one experimental study supports the notion that placebo-like beliefs tend to rely on stronger priors than new information in the context of sensory processing (Baker, Gamer, Rauh, & Brassen, 2022). On the other hand, some researchers argue that delusionality emerges from a *lack* of strong priors – that is, priors that are overly flexible, plastic, and updatable, such that they are easily overridden through new experiences. Some (Haarsma et al., 2020; Powers et al., 2025) have postulated that weak priors are characteristic of earlier phases of psychosis, including psychosis-risk syndromes, when beliefs are malleable, allowing new delusions to emerge (a state sometimes called ‘delusional mood’). Furthermore, certain theoretical accounts suggest that hallucinations are linked to *greater* weighting of sensory evidence and *lower* emphasis on prior beliefs (reduced prior-to-likelihood ratio; Weilnhammer et al., 2020).

Per PC theory, paranoia can *also* be explained in terms of strongly weighted priors and weaker likelihoods that theoretically result in individuals taking more time to integrate new information and develop beliefs related to placebo and nocebo responses (Hird et al., 2023). Paranoia is also thought to relate closely to expectations of volatility, which have a strong influence on learning rates and the updating of priors, especially in emotionally salient experiences (Sheffield et al., 2022). Thus, an individual experiencing these or similar traits may consistently overestimate environmental volatility in part due to worry and anxiety stemming from the stimuli (Sheffield et al., 2022). With regard to antipsychotic medications, an individual with paranoid cognitions may be less likely to have positive expectations about medication or placebo, which may produce a nocebo effect and, subsequently, treatment noncompliance. Over time, however, the weight of likelihoods may slowly increase if the individual experiences benefits from the medication. As learning mechanisms work to reduce prediction errors, placebo beliefs may become further integrated into an individual’s model of the world (Hird et al., 2023).

## Aberrant salience attribution and placebo effects

Another critical aspect of belief formation thought to play a role in delusion formation is salience attribution. According to the aberrant salience hypothesis, individuals with psychosis inadvertently assign undue value or importance to seemingly mundane stimuli or experiences (Kapur, 2003). In essence, this hypothesis considers how aberrant learning and conditioning contribute to salience attribution, laying the foundation for the development of delusions or other odd beliefs. As aberrant salience influences where an individual places a focus or an emphasis on incoming stimuli, this process may be representative of an attempt to detect a signal in noisy input. In the absence of strong priors, this can lead to maladaptive salience attribution and the formation of delusional beliefs, as individuals are attempting to fit perceptions and events to specific prior beliefs (Petrovic & Sterzer, 2023). The aberrant salience hypothesis is grounded in anomalous learning, resulting in mundane stimuli and events being labeled as biologically significant (Kapur, 2003). These aberrant attributions of salience to experience can, through conditioning, influence expectations and contribute to the development of odd or delusional beliefs (Petrovic & Sterzer, 2023) and by extension, placebo and nocebo effects. In other words, experiences of aberrant salience influence expectations and learning experiences (Kapur, 2003; Waltz & Gold, 2016). Critically, the concept of salience attribution is closely tied to ideas regarding the functional roles of dopamine systems in the brain, with excessive/erratic dopamine system activity either causing or resulting from maladaptive attributions of salience (Whitton, Treadway, & Pizzagali, 2015). Different SSD symptoms (positive versus negative) may be associated with different abnormalities in salience attribution (excessive versus insufficient), brought on by different disruptions of dopamine system function (hyperactivity versus hypoactivity; Whitton, Treadway, & Pizzagalli, 2015). According to the aberrant salience hypothesis, antipsychotic medications are ‘antipsychotic’ because they reduce the salience attributed to mundane or insignificant stimuli and thereby attenuate positive symptoms (Kapur, 2003).

## Placebo responses in RCTs for adjunctive medication for SSDs

Treatments for symptoms that are grounded in beliefs and expectations may be more subject to placebo effects than treatments for negative (Fraguas et al., 2019) and cognitive symptoms (Keefe et al., 2017). In fact, Czobor et al. (2022) have suggested that negative symptom placebo effect estimates are smaller than previously reported, due to the stability and lack of variability of negative symptoms over time. This may also be highlighted in Keefe et al.’s (2017) findings of no strong placebo effects for cognition in those with schizophrenia. The adaptive attribution of salience is also critical to the refinement of goal-directed behavior, as motivational deficits are thought to arise from a blunted ability to properly assign incentive value to stimuli and events (Kapur, 2003; Waltz & Gold, 2016).

The influence of placebo responses in SSDs has also been examined in the context of clinical trials for medications designed to enhance motivation and cognition, though not all have used placebo groups. Findings from these studies generally indicate that placebo responses in such trials are considerably smaller than they are in trials of antipsychotic medications. In a meta-analysis of 12 randomized placebo-controlled RCT studies, involving 813 patients with schizophrenia, Keefe et al. (2017) found that, in clinical trials for candidate compounds for cognition enhancement, placebo responses were very small, suggesting that placebo effects for cognition in schizophrenia are virtually nonexistent. Clinical trials of the add-on use of arginine vasopressin for learning and memory in SSDs revealed treatment responses that exceed placebo responses (Geng et al., 2017). The use of modafinil for cognition appears to have neither a strong placebo nor treatment effect in people with SSDs (Kumar, 2008), although Strzelecki et al. (2016) observed improvements in cognition in patients with SSD administered modafinil, but not in the placebo group. Regarding social functioning in patients with schizophrenia, both placebo and treatment responses were observed in an RCT for paliperidone ER (Patrick et al., 2010). Finally, placebo effects associated with the use of various other adjunctive or repurposed medications remain to be clearly elucidated (Cao et al., 2021; Correll et al., 2023).

## Future directions

Because the placebo effects of antipsychotic and other medications in SSDs have rarely been examined in a mechanistic manner, their underlying physiology remains unknown. Based on the theories, concepts, and gaps in the literature relevant to understanding the placebo effect in SSDs, identified by us and others, there are several possible future directions that the field may take (Table 1).

**Table 1.** Future directions for placebo research in SSDs

Future directions	Examples
Investigating types of placebo effects through clinical trials	Assessing placebo effects for different types of medications Assessing cultural and demographic influences Assessing expectations in clinical trials Exclusion of non-help-seeking individuals in RCTs Age of participants
Investigating contextual factors in experimental studies	Assessing placebo effects for different conditions Evaluating subjective and objective measures Manipulating expectations Assessing insight and cognitive functioning
Application of novel research methods	Virtual reality Computational psychiatry Advanced statistics Experimental conditioning

When examining placebo responses in clinical trials involving individuals with psychosis, it is essential to consider contextual and methodological differences compared to other clinical populations (Atlas, 2023). Expectations shaped by prior learning, beliefs, uncertainty, and reward sensitivity should be assessed in both pharmacological and nonpharmacological RCTs. These variables, alongside insight, symptoms, and cognitive functioning, should be included as covariates to better understand the magnitude of placebo and nocebo effects. However, expectations are often symptom-dependent in psychosis, making it difficult to control for them independent of symptom severity (Murray & Stoessl, 2013). Disparities related to culture, sex, gender, and socioeconomic status also influence expectations and treatment engagement, potentially leading to underrepresentation of certain groups in placebo research (Cundiff-O’Sullivan et al., 2023; Jarvis et al., 2020). Sex/gender differences in SSDs, such as earlier age of onset and more severe negative symptoms in men, further justify examining biological sex as a moderator of placebo effects (Giordano, Bucci, Mucci, Pezzella, & Galderisi, 2021; Shafir, Olson, & Colloca, 2022). While antipsychotic placebo effects in adults have been explored (Kalaria et al., 2019, 2020), there is limited research in children and adolescents.

Applying methods common in non-industry-sponsored trials, such as prioritizing knowledge of participants’ histories, attention to quality and detail, and the reduction of assessment bias, may be possible options to reduce industry pressures in future trials (Fraguas et al., 2019). One recommendation is to create networks of academic centers to match the recruitment and output of industry. For example, there is now a large network of academic centers and sites in Europe that work together to promote, centralize, and coordinate schizophrenia research called the European Group for Research in Schizophrenia (Galderisi, 2023). The creation of such a network in North America would similarly benefit future research and reduce industry pressures.

One important factor to consider is differences in outcome measures (i.e., subjective measurements of symptom ratings versus objective cognitive performance scores). Although objective markers of positive and negative symptoms are lacking, emerging tools such as digital phenotyping and neuroimaging (e.g. conditioned hallucinations [Powers et al., 2017] and geolocation [Horan et al., 2024]) may help to quantify constructs such as paranoia, hallucinations, anhedonia, and avolition. In examining the convergence and divergence of subjective and objective measurements, we may bolster our understanding of placebo responses in people with SSDs. Experimental studies designed specifically for people with SSDs and medications of interest are needed, given that most placebo findings stem from RCTs primarily focused on treatment efficacy (Kinon et al., 2011; Murray & Stoessl, 2013). It remains unclear whether placebo responses for psychiatric symptoms generalize to other conditions, such as pain. For example, predictions regarding placebo analgesia in psychosis may not be straightforward, as certain individuals with schizophrenia experience higher sensitivity to acute pain and lower sensitivity to prolonged and chronic pain (called the ‘Pain Threshold Paradox’; Lévesque et al., 2012; Nagamine, 2023).

Designs involving the *manipulation* of expectations may clarify the role of belief valence. Interventions targeting appraisals and expectations have been shown to reduce negative symptoms (Fraguas et al., 2019). Mechanisms such as social learning and verbal suggestion can shape placebo responses, with evidence from pain modulation and emotion regulation studies (e.g., Schenk et al., 2017; Shafir, Israel, & Colloca, 2023). Eliciting positive expectations through social interactions has also improved mood in placebo-controlled trials (Baker et al., 2022) and brief psychoeducation via a Placebo-Control Reminder Script reduced depressive symptoms in individuals with psychosis or depression diagnoses (Cohen et al., 2021). Theoretical models, such as predictive coding, have been successfully applied to placebo analgesia (Büchel et al., 2014; Ongaro & Kaptchuk, 2019) but rarely to psychotic or depressive symptoms. Investigating placebo and nocebo effects in SSDs may yield insights into alternative mechanisms for symptom relief, especially considering the side effects of psychiatric medications.

Future research should employ novel designs and analytic methods – including virtual health tools, digital phenotyping, expectation conditioning, and emotion regulation-based paradigms – to isolate placebo mechanisms. Technology such as artificial intelligence-based machine learning algorithms, remote assessments, and virtual reality can be applied to identify characteristics of placebo responders, though future research to confirm proof of concept is needed (Horan et al., 2024). Utilizing advanced statistics such as mixed-effects models, stimulations, dropout models, and growth mixture modeling with maximum-likelihood estimations can refine our understanding of placebo-drug differences (Kozielska et al., 2013; Reddy et al., 2011, 2012, 2013). Additionally, nonpharmacological interventions such as psychosis therapies and transcranial magnetic stimulation show promise and warrant further placebo-related investigation (Paillère‐Martinot et al., 2017; Turkington et al., 2017), as do repurposed medications with potential placebo effects (Correll et al., 2023).

## Questions and concerns regarding placebo responses in RCTs involving antipsychotic medications

Despite the clinical relevance (Alphs et al., 2012; Hird et al., 2023), longitudinal trends in antipsychotic placebo effects are rarely evaluated. Evidence suggests that placebo participants tend to relapse over time, reverting to baseline symptom levels (Ceraso et al., 2020; van de Laar et al., 2001). It is also unknown whether placebo responses in SSDs generalize or vary across treatment types, such as antipsychotic, analgesic, or antihypertensive medications. Given the chronic nature of SSDs and the side effects of antipsychotic medications that contribute to elevated morbidity (Peritogiannis et al., 2022), understanding the sustainability of placebo responses in SSDs and their relation to treatments is valuable (Ali et al., 2023; Ceraso et al., 2020). Ethical concerns are especially salient in placebo trials for psychosis, given the vulnerability present in this population (Melamed et al., 2009). For example, the duration of placebo arms in first-episode psychosis trials should be minimized to avoid extended untreated psychosis (Haddad & Correll, 2018), although placebo controls may help avoid exposing patients to the adverse effects of ineffective medications (Oliveira et al., 2009). Ultimately, focusing on theoretical constructs such as predictive coding and aberrant salience provides testable frameworks for advancing placebo research in SSDs.
