# Placebo and Nocebo Phenomena in Schizophrenia Spectrum Disorders: A Narrative Review on Current Knowledge and Potential Future Directions – CORRIGENDUM

**DOI:** 10.1017/S0033291726103493

**Published:** 2026-02-02

**Authors:** Sherry D. Pujji, Luana Colloca, James A. Waltz

**Affiliations:** 1 Maryland Psychiatric Research Center (MPRC), Department of Psychiatry, University of Maryland School of Medicine, Baltimore, MD, USA; 2 Department of Pain and Translational Symptom Science, Placebo Beyond Opinions (PBO) Center, University of Maryland School of Nursing, Baltimore, MD, USA

When this article was originally published in Psychological the author list was in an incorrect order. This has been updated in the original article.

The authors apologise for this error.
